# Case Report: Glioblastoma in cohabiting partners: a molecularly characterized temporal cluster

**DOI:** 10.3389/fonc.2026.1890635

**Published:** 2026-07-28

**Authors:** Evelyn Gisell Belotti, Nicholas Giulio Raccagni, Arianna Barbotti, Francesco DiMeco, Ignazio Gaspare Vetrano

**Affiliations:** 1 Department of Neurosurgery, Fondazione IRCCS Istituto Neurologico Carlo Besta, Milan, Italy; 2 Department of Oncology and Hematology-Oncology, Università degli Studi di Milano, Milan, Italy; 3 School of Medicine and Surgery, University of Milano-Bicocac, Milan, Italy; 4 Department of Neurological Surgery, Johns Hopkins Medical School, Baltimore, MD, United States; 5 Department of Biomedical Sciences for Health, Università degli Studi di Milano, Milan, Italy

**Keywords:** case report, cohabitation, exposure assessment, giant cell glioblastoma, glioblastoma

## Abstract

**Background:**

Glioblastoma is the most lethal primary brain tumor of adults, with an age-standardized incidence of 5–7 per 100,000 person-years and a median survival of approximately 15 months despite maximal safe resection, radiotherapy, and temozolomide. Its occurrence in two unrelated cohabiting individuals within a short interval is an uncommon event that inevitably raises the question of shared causation, one that available retrospective methods rarely resolve.

**Case presentation:**

Two unrelated adults from northern Italy, partners sharing a household since 2018 and a workplace since 2016, developed glioblastoma approximately 20 months apart. Case 1 was a 47-year-old woman with giant cell glioblastoma, IDH-wildtype, central nervous system (CNS) WHO grade 4, O^6^-methylguanine-DNA methyltransferase (MGMT) promoter unmethylated and telomerase reverse transcriptase promoter (pTERT)-negative. She developed recurrence with gliosarcomatous features and acquired homozygous cyclin-dependent kinase inhibitor 2A/B (CDKN2A/B) deletion, and died following craniospinal leptomeningeal dissemination. Case 2 was a 51-year-old man with biopsy-proven glioblastoma, IDH-wildtype, CNS WHO grade 4, meeting WHO 2021 criteria on both histologic grounds (focal necrosis) and molecular grounds (pTERT C250T mutation).

**Exposure assessment:**

A structured retrospective exposure-history questionnaire was administered to Case 2 during his diagnostic hospital admission. The assessment documented a shared office-based occupational and residential context without identifying a specific carcinogenic exposure. Multiple relevant domains, including radon, indoor air quality, household and water chemistry, remained unmeasured and should not be interpreted as negative findings.

**Conclusion:**

This report documents a temporal cluster without implying shared causation. Its primary contribution is the detailed molecular characterization of both cases, including recurrence profiling in Case 1, and a transparent, reproducible exposure-history framework that distinguishes absent from unmeasured data.

## 1 Introduction

Glioblastoma is the most common malignant primary brain tumor of adults, with a median overall survival of approximately 15 months under standard chemoradiotherapy (1–4). Despite decades of investigation, ionizing radiation remains the sole environmental exposure consistently linked to its development; evidence implicating pesticides, solvents, non-ionizing radiation, and other environmental agents has been inconsistent and not replicated at a level sufficient to modify practice (5, 6).

Against this background, glioblastoma arising in two unrelated cohabiting individuals is a rare event that raises a question of shared causation (7–9). A handful of such reports have appeared over the past four decades, none identifying a reproducible shared carcinogen. These cases resist easy interpretation: they may reflect chance coincidence, a shared but undetected exposure, a common susceptibility, or some combination of factors that current retrospective methods cannot resolve. What is consistently lacking is a framework for documenting what was assessed, what was found and, crucially, what remained unmeasured.

We report two unrelated adults who shared a household since 2018 and a workplace since 2016, and who developed IDH-wildtype glioblastoma approximately 20 months apart. We describe the clinical, imaging, histopathologic, and molecular features of both cases, including extended recurrence profiling in Case 1, and present a structured retrospective exposure assessment. The report was prepared in accordance with the CARE case-report guidelines (10). Beyond the two cases themselves, the principal contribution we wish to advance is methodological: a standardized, reproducible framework for retrospective exposure assessment that explicitly separates exposures that were assessed and found absent from those that were never measured. We propose this framework as a practical instrument to be applied whenever clinicians and researchers encounter suspected glioma clusters, so that future reports can be compared on common terms rather than assembled *ad hoc*.

## 2 Case presentation

### 2.1 Methods of case ascertainment and exposure reconstruction

Clinical, imaging, histopathologic, and molecular data were retrospectively extracted from available medical records. A structured exposure-history questionnaire (**Supplementary Table 1**) was administered to Case 2 during his diagnostic hospital admission preceding stereotactic biopsy. The respondent provided information for himself and, to the extent known to him, for his deceased partner. No direct environmental measurements were available; the assessment constitutes a structured historical reconstruction, not a formal environmental or occupational hygiene investigation.

### 2.2 Case 1

**Figure 1:**
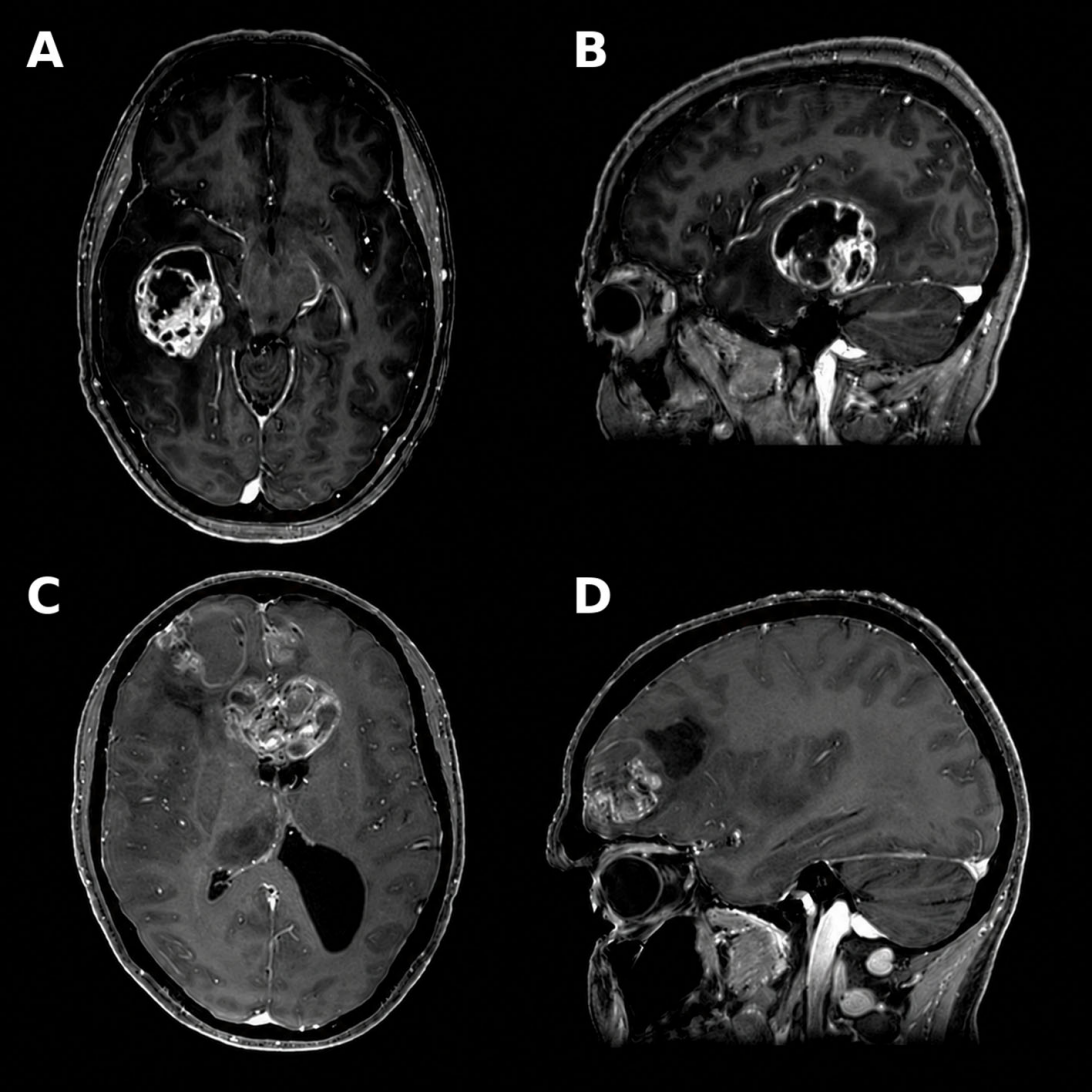
*Preoperative brain MRI.* **(A, B)** Case 1: axial and sagittal post-contrast T1-weighted images showing a right temporal enhancing lesion with cystic-necrotic features, consistent with giant cell glioblastoma. **(C, D)** Case 2: axial and sagittal post-contrast T1-weighted images showing a right fronto-insulo-temporal intra-axial enhancing lesion with corpus callosum involvement, mass effect, and contralateral extension.

In November 2023, a 47-year-old woman was referred after a 10-day evolution of progressive holocranial headache, worse in the supine position, accompanied by gait instability and right-hand paresthesias. Examination revealed mild left upper-limb pronation and an unstable gait. Brain MRI demonstrated a right temporal intra-axial enhancing lesion with cystic-necrotic features (**Figures 1A, B**).

On November 2023, she underwent right temporal craniotomy with neuronavigation-guided resection. Histopathologic examination established a diagnosis of giant cell glioblastoma, IDH-wildtype, CNS WHO grade 4 (11, 12). Immunohistochemistry demonstrated GFAP, p53, and PTEN positivity; IDH1 R132H and EGFR were negative. IDH-wildtype status was confirmed by PCR and Sanger sequencing of IDH1 exon 4 and IDH2 exon 4. Mismatch repair proteins (MSH2, MLH1, PMS2, MSH6) showed intact nuclear expression; PD-L1 was positive in fewer than 1% of neoplastic cells; ATRX assessment was subsequently incorporated into the recurrence molecular panel. TERT promoter (pTERT) mutation testing by PCR and Sanger sequencing was negative; MGMT promoter methylation was absent by methylation-specific PCR (quantitative value 0.04; methylation threshold ≥0.1). EGFR amplification was not assessed at initial surgery; non-amplification was confirmed at recurrence molecular profiling. The postoperative course was complicated by transient mild dysarthria; at discharge, mild left homonymous hemianopia persisted.

She received radiotherapy with concomitant and adjuvant temozolomide according to the Stupp regimen (60 Gy in 30 fractions with concurrent temozolomide 75 mg/m² daily; followed by adjuvant temozolomide 150–200 mg/m² for 5 days per 28-day cycle) (13), completed in February 2024; she went on to receive six adjuvant temozolomide cycles. She was subsequently enrolled in the ClinGlio study (NCT04250922) (14), from which she withdrew in March 2024 following imaging changes meeting protocol withdrawal criteria. Recurrence was confirmed on follow-up MRI in September 2024, prompting discontinuation of adjuvant temozolomide.

On October 2024, she underwent repeat right temporal resection. Histopathology confirmed secondary gliosarcoma, recurrent giant cell glioblastoma, IDH-wildtype, CNS WHO grade 4, with diffuse gliosarcomatous features and focal chondroid differentiation. Extended molecular profiling at recurrence demonstrated: loss of heterozygosity (LOH) at 9p, 10q, 17p, and 19q; acquired homozygous deletion of CDKN2A and CDKN2B (absent at initial diagnosis); heterozygous deletion of PTEN and PDGFRA; BRAF V600E negative; pTERT negative (confirmed); EGFR non-amplified (confirmed); MGMT promoter unmethylated (confirmed). The acquired CDKN2A/B homozygous deletion, in the context of extensive LOH, documents clonal selection under therapeutic pressure, a pattern characteristic of secondary gliosarcoma (15). She was subsequently readmitted with fever, somnolence, and psychomotor slowing; brain and spine MRI together with cerebrospinal fluid cytology supported craniospinal leptomeningeal dissemination. A wound-fluid culture grew Pseudomonas aeruginosa; the superficial wound infection was managed with targeted antimicrobials. Given her rapidly declining performance status and the diffuse leptomeningeal spread, no further tumor-directed therapy was deemed appropriate, and she received best supportive care. She died of leptomeningeal disease progression in November 2024.

### 2.3 Case 2

In July 2025, a 51-year-old man, the surviving partner of Case 1, presented with approximately one month of progressive personality change, persistent headache, vertigo, and nausea. On direct questioning, personality changes with apathy and social withdrawal had in fact begun two to three months earlier, initially attributed to grief following his partner’s recent death. Neurological examination on admission was unremarkable. Brain MRI showed a right fronto-insulo-temporal intra-axial lesion with corpus callosum involvement, mass effect, midline shift, and contralateral extension (**Figures 1C, D**).

The right fronto-insulo-temporal location with corpus callosum involvement precluded oncologically meaningful resection; following multidisciplinary team discussion, stereotactic needle biopsy was performed. On August 2025, all 16 sampled fragments were non-fluorescent on intraoperative 5-aminolevulinic acid (5-ALA) guidance (16). 5-ALA non-fluorescence does not exclude tumor: the negative predictive value for the absence of tumor tissue in non-fluorescent fragments is only 38% (17), meaning the majority still contain tumor cells, but from the infiltrative margin rather than the metabolically active core. This is consistent with the low neoplastic cellularity (20%) and focal Ki-67 of 7–8%.

Histopathologic examination revealed fragmented nervous tissue partially infiltrated by a cellular neoplasm composed of hyperchromatic, pleomorphic nuclei with mitotic figures and focal areas of necrosis. Immunohistochemistry demonstrated GFAP and ATRX positivity, partial Olig2 and p53 positivity, negative IDH1 R132H staining. Molecular testing demonstrated pTERT C250T mutation, absence of IDH1/2 hotspot mutations, MGMT promoter unmethylation, and heterozygous CDKN2A/B deletion. EGFR was not amplified by TaqMan PCR (copy number 1.158; threshold ≥4); the IHC for EGFR was non-evaluable. The differential diagnosis included primary CNS lymphoma and metastatic disease, both excluded by the immunohistochemical profile and clinical staging.

The integrated diagnosis was glioblastoma, IDH-wildtype, CNS WHO grade 4, supported independently by both the histologic criterion (focal necrosis) and the molecular criterion (pTERT C250T) under WHO 2021 and cIMPACT-NOW criteria (11, 18), with the caveat that biopsy-limited sampling precluded complete morphological characterization. Referral for adjuvant neuro-oncology and radiotherapy was planned; however, despite an unremarkable neurological examination on admission, he underwent rapid neurological deterioration in the weeks following the biopsy, consistent with the extensive, biologically aggressive, and surgically unresectable tumor burden, and he died in August 2025, before initiating treatment. Complete clinical and molecular data for both cases are summarized in **Table 1**.

**Table 1:** Clinical, histopathologic, and molecular features of both cases.

Feature	Case 1	Case 2
Age/sex	47-year-old woman	51-year-old man
Symptom onset	10-day history of progressive headache, gait instability, right-hand paresthesias	~1 month of personality change, persistent headache, vertigo, nausea
Tumor location	Right temporal	Right fronto-insulo-temporal; corpus callosum involvement; midline shift; contralateral extension
Surgical procedure	Right temporal craniotomy (Nov 2023); repeat resection at recurrence (Oct 2024)	Stereotactic needle biopsy (Aug 2025); 5-ALA: all 16 fragments non-fluorescent
Integrated diagnosis	Giant cell glioblastoma, IDH-wildtype, CNS WHO grade 4	Glioblastoma, IDH-wildtype, CNS WHO grade 4 (focal necrosis + pTERT C250T, each independently sufficient under WHO 2021/cIMPACT-NOW)
MGMT promoter	Unmethylated (MSP; quantitative value 0.04; threshold ≥0.1)	Unmethylated (MSP)
IDH status	IDH-wildtype (IHC + Sanger sequencing IDH1 exon 4 and IDH2 exon 4)	IDH-wildtype (IHC + IDH1/2 hotspot sequencing)
pTERT	NEGATIVE (PCR + Sanger; consistent with giant cell GBM molecular subtype)	C250T mutation (fulfills molecular WHO 2021 GBM criterion independently)
EGFR	Negative (IHC); non-amplified confirmed at recurrence	Non-evaluable (IHC); not amplified (TaqMan PCR; copy number 1.158; threshold ≥4)
CDKN2A/B	Not tested at initial diagnosis (see recurrence panel)	Heterozygous deletion
+7/−10	Not assessed	Not assessed
IHC profile	GFAP+, p53+, PTEN+, IDH1−, EGFR− (IHC) MMR proteins: intact PD-L1: <1% neoplastic cells	GFAP+, ATRX+, Olig2 partial+, p53 partial+, IDH1 R132H− Ki-67: ~7–8% focal (20% neoplastic cellularity)
Disease course	Recurrence with gliosarcomatous transformation; craniospinal leptomeningeal dissemination	Died August 2025, before initiating adjuvant treatment
Outcome	Deceased	Deceased
Recurrence (Case 1 only) Oct 2024	Secondary gliosarcoma, IDH-wildtype, WHO grade 4; focal chondroid differentiation LOH: 9p, 10q, 17p, 19q CDKN2A/B: homozygous deletion (ACQUIRED) PTEN/PDGFRA: heterozygous deletion BRAF V600E: negative; pTERT: negative; EGFR: non-amplified; MGMT: unmethylated	N/A

### 2.4 Structured exposure assessment

**Figure 2:**
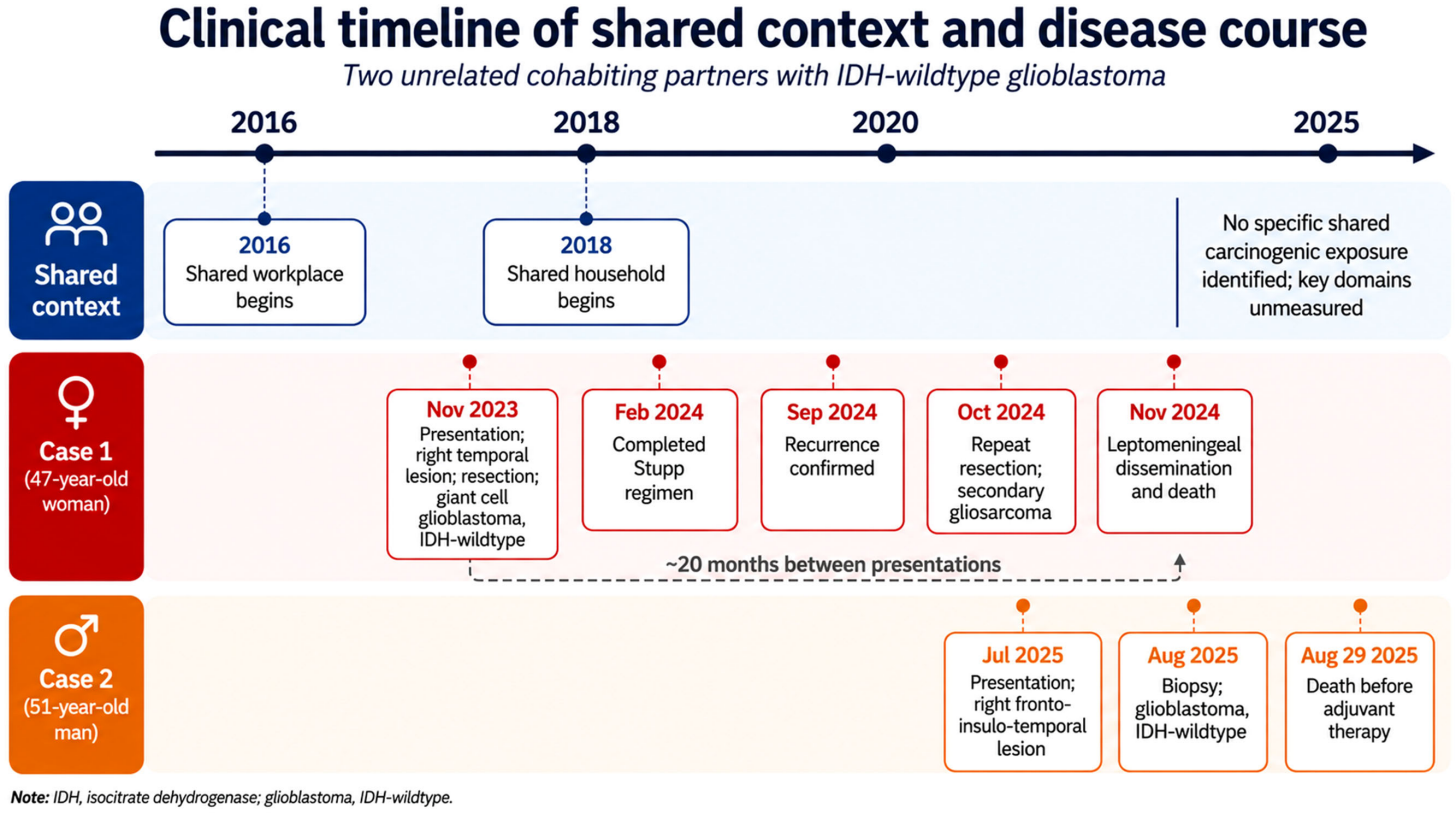
Clinical timeline of shared context and disease course.

The two patients had worked at the same office-based administrative workplace since 2016 and shared a rented household since 2018 (**Figure 2**). Occupational exposures were characteristic of an administrative setting: no contact with industrial processes, pesticides, ionizing radiation, heavy metals, or recognized glioma-associated solvents was documented, and no formal occupational hygiene assessment was available. Their shared residence had reportedly undergone renovation in 2015 and was heated by radiators; no household activities or materials clearly associated with a shared carcinogenic risk were identified.

External sources near the residence included a municipal waste-to-energy facility (operational since the early 2000s) and a municipal landfill. Groundwater contamination in the immediate vicinity of the waste-to-energy site, including mercury at concentrations reported to exceed regulatory thresholds sixfold, and chlorinated organic compounds above contamination threshold concentrations, was the subject of a formal municipal inquiry in early 2025 (19). Epidemiological evidence on residential proximity to waste incineration facilities and glioma risk is inconsistent; no individual-level water testing, biomonitoring, or air-quality measurement was available for either patient (20). In particular, ambient ultrafine and combustion-derived nanoparticles were not measured near the residence or the implicated facility. Such particles can translocate to the central nervous system, in part via the olfactory and trigeminal pathways, as demonstrated in both animal models and human autopsy studies (21, 22), and emerging, though still inconclusive, epidemiological evidence has linked ambient ultrafine particle exposure to the incidence of brain tumors, including the more aggressive gliomas (23). We list this as an unmeasured domain rather than a negative finding. The detailed findings of the municipal groundwater inquiry, and any associated environmental monitoring data, were not available to us and could not be incorporated into this report.

Family history included selected non-CNS malignancies in both patients but no hereditary glioma syndrome, and germline testing was not performed (24). Lifestyle exposures were partially discordant: one patient was an active smoker, the other had passive smoke exposure only. No radon measurement, indoor air quality assessment, household water analysis, biomonitoring, or formal electromagnetic-field study was available. The retrospective assessment did not identify a specific shared carcinogenic exposure. Critically, multiple domains remained unmeasured and cannot be interpreted as negative. A structured summary of all assessed domains, distinguishing shared findings, individual or discordant findings, and unmeasured domains for both patients, is presented in **Table 2**; the complete questionnaire administered during case ascertainment is provided in full in **Supplementary Table 1**.

**Table 2:** Structured retrospective exposure assessment for both patients, summarizing, for each domain, what was assessed, shared findings, individual or discordant findings, and domains that were not measured or unavailable.

Domain	What was assessed	Shared findings	Individual/discordant findings	Not measured/unavailable
Cohabitation and work	Residential addresses (preceding 30 years), cohabitation duration and any separations, workplace, job tasks	Shared workplace (2016–); shared household (2018–); office-based administrative setting	Different roles within the same agency	No quantitative workplace exposure assessment
Housing	House type, construction year and materials, maintenance, mold/humidity, heating and fuel, radon, water	Rented house; renovation 2015; radiator heating	None specifically documented	No radon testing; no indoor-air, mold, or building-materials assessment
Environmental context	External pollution sources, indoor/outdoor air quality, drinking-water analyses, EMF sources, radon	Proximity to a waste-to-energy plant and municipal landfill; publicly discussed groundwater-contamination concern (unverified at individual level)	None specifically documented	No individual-level water, soil, air, ultrafine-particle, or toxicologic measurements
Occupational	Complete work history (preceding 30 years), documented toxicant exposure, protective equipment, workplace EMF	Shared office setting; no glioma-associated toxicants documented	Male: administrative role (2005–); female: administrative role (2016–)	No industrial hygiene or formal EMF assessment
Lifestyle	Diet, alcohol, active and passive smoking, physical activity, travel, animal contact	Similar diet; shared travel destinations	Active smoker (female) vs passive smoke exposure (male); differing alcohol and physical-activity patterns	No biomonitoring data
Clinical history	Previous tumours or neurologic disease, prior diagnostic/therapeutic radiation, chronic disease, regular medications	No prior therapeutic or diagnostic radiation reported in either patient	Distinct comorbidity and medication profiles	No systematic dosimetry of prior diagnostic imaging
Family/genetic	Family cancers and age at diagnosis, hereditary tumour syndromes, genetic testing, family neurologic disease	No hereditary glioma syndrome; no family neurologic history	Non-CNS malignancies in the family history of both patients	No germline testing performed in either patient

The complete questionnaire is provided in **Supplementary Table 1**.

Timeline summarizing the shared workplace and household history of the two unrelated cohabiting partners and the main clinical milestones of both glioblastoma cases. The interval between presentations was approximately 20 months.

## 3 Discussion

Both tumors fulfil contemporary diagnostic criteria for glioblastoma, IDH-wildtype, CNS WHO grade 4, but they show divergent histomolecular profiles. Case 1 was a giant cell glioblastoma with unmethylated MGMT promoter and pTERT-wildtype status, followed by recurrence with gliosarcomatous transformation and acquired homozygous CDKN2A/B deletion. Case 2 was a biopsy-diagnosed glioblastoma with focal necrosis, unmethylated MGMT promoter, pTERT C250T mutation, and heterozygous CDKN2A/B deletion. Distinct molecular profiles do not exclude a shared exposure or susceptibility, because glioblastoma tumorigenesis is molecularly heterogeneous and environmental carcinogenesis may operate through mutational, epigenetic, microenvironmental, or selective mechanisms that do not necessarily produce identical molecular end points in different individuals.

This distinction is important because the inference of environmental causality from tumor molecular profiles generally requires more than targeted diagnostic markers. Whole-genome analyses have shown that cancer genomes often contain multiple superimposed mutational signatures reflecting endogenous processes, exogenous exposures, defective DNA-maintenance mechanisms, and processes of unknown cause (25). Experimental exposure studies further show that DNA damage induced by environmental agents can be resolved by different repair and/or replicative pathways, producing an assortment of mutational outcomes even for a single agent (26). The tobacco-smoking literature provides an illustrative example: a well-established carcinogenic exposure is associated with increased burdens of multiple distinct mutational signatures that contribute to different extents across different cancer types (27). Therefore, pTERT-wildtype status in one tumor and pTERT mutation in the other cannot be used to exclude a common exposure; conversely, in the absence of whole-genome sequencing and mutational-signature analysis, these findings cannot substantiate one either. Environmental carcinogenesis may also involve non-mutational or tumor-promoting mechanisms, which further limits etiologic inference from a restricted diagnostic molecular panel (28).

The pTERT status of the two tumors is best interpreted within the known biological spectrum of IDH-wildtype glioblastoma. TERT promoter mutations are among the most frequent alterations in adult glioblastoma but they are not universal (29). Case 2 shows a common molecular feature of conventional adult IDH-wildtype glioblastoma, whereas the pTERT-wildtype status of Case 1 is compatible with the reported heterogeneity of giant cell glioblastoma, a rare IDH-wildtype variant characterized by enrichment for TP53 and ATRX alterations, lower EGFR amplification frequency, and lower TERT promoter mutation frequency (30).

Similarly, the recurrence of Case 1 with gliosarcomatous transformation and acquired homozygous CDKN2A/B deletion should be framed as evidence of tumor evolution rather than as evidence bearing directly on the shared-exposure hypothesis. The acquisition of CDKN2A/B homozygous deletion and multiple LOH events at recurrence is consistent with reported clonal evolution from primary glioblastoma to secondary gliosarcoma (15).

The temporal proximity of the two diagnoses, approximately 20 months in two individuals sharing household and workplace, is uncommon against a background incidence of 5–7 per 100,000 person-years, but its rarity is difficult to quantify meaningfully from a single ascertained pair. Any such estimate is highly sensitive to assumptions regarding age-specific incidence, sex, population structure, and the *post hoc* ascertainment bias that is intrinsic to cluster reports (5, 6). (see **Supplementary Table 2**).

From an epidemiological perspective, the shared household and workplace history justifies a structured exposure assessment, but the present data do not identify a specific carcinogenic exposure. Ionizing radiation remains the best-established environmental risk factor for glioma, whereas evidence for pesticides, N-nitroso compounds, air pollution, solvents, and radiofrequency electromagnetic fields remains inconsistent or insufficient for causal attribution in individual cases (5, 6, 31, 32). The contextual environmental features documented in this case, residential proximity to a waste-to-energy facility with documented groundwater contamination, are noteworthy and represent domains that warrant individual-level measurement in future investigations; however, the absence of such measurements precludes any etiological inference in this case. Consistent evidence linking residential proximity to incineration facilities with glioma risk has not been established (20).

The cohabiting glioma literature spans four decades, lacks molecular classification by current standards in most reports, and has identified no reproducible shared exposure (7–9). The Fehr and Auer case, involving divorced individuals who had not cohabited for 20 years, is categorically distinct from cohabiting pairs (33). Population-based studies have not demonstrated an excess of brain tumors risk among spouses of affected patients (34, 35).

It is therefore useful to weigh explicitly what favors and what argues against shared causation in this pair. In favor are the prolonged shared residential and occupational environments, the documented environmental hazards in the immediate vicinity of the residence, and the temporal clustering of two diagnoses of an uncommon tumor within approximately 20 months. Against a shared deterministic cause are the divergent histomolecular profiles of the two tumors, the fact that both patients were within the typical age range for sporadic IDH-wildtype glioblastoma, the discordant individual exposures such as smoking status, the absence of any hereditary glioma syndrome or demonstrated germline susceptibility, and the lack of population-level evidence for an excess of brain tumors among cohabiting partners. On balance, the available data are compatible with chance concurrence, with a shared but unmeasured exposure, or with independently arising sporadic tumors, and do not allow these possibilities to be distinguished.

Direct interpersonal transmission of tumor cells, whether as intact cells or via tumor-derived extracellular vesicles, is not a plausible mechanism here: naturally transmissible cancers are confined to a few non-human species (36), allogeneic tumor cells are efficiently rejected in immunocompetent humans, and although the horizontal transfer of mutant genes by tumor-derived extracellular vesicles has been described experimentally, it is constrained by barriers that render transformation of normal recipient cells transient or absent (37, 38); the divergent molecular profiles of the two tumors are in any case incompatible with a single transmitted clone. The second mechanism is a shared oncogenic infection. A role for human cytomegalovirus in glioma pathogenesis has been proposed but remains controversial and unproven, with conflicting detection results and, at most, a putative oncomodulatory rather than oncogenic role (39). A shared infectious history was not captured by the exposure questionnaire and could not be reconstructed; we therefore regard it, like the environmental domains above, as unmeasured rather than excluded.

The principal and most generalizable contribution of this report is the structured exposure-assessment framework itself, which we propose as a template for the investigation of future suspected glioma clusters. The methodological contribution of this report lies in the distinction between not identified and excluded. Radon, indoor air quality, household water chemistry, soil contamination, biomonitoring, and germline susceptibility were not measured at the individual level; they are undocumented, not demonstrably absent. Future reports of suspected glioma clusters should specify the circumstances of exposure reconstruction, integrate current molecular classification, and enumerate unmeasured domains explicitly rather than by omission. The present pair is therefore best interpreted as a temporal cluster of two IDH-wildtype glioblastomas with divergent histomolecular profiles. In plainer terms, the two tumors do not look like two copies of one underlying process, and this makes a single shared molecular cause unlikely; but a difference in molecular profile is not proof of a difference in cause, because the same exposure can leave different molecular marks in different people. The molecular findings therefore weaken the idea of a simple common trajectory without ruling out a shared exposure, a shared susceptibility, or simple coincidence.

## 4 Limitations

This report has important limitations. Exposure reconstruction was retrospective and based on a questionnaire completed by the surviving patient, who provided information for himself and, where known, for his deceased partner; a directly completed structured interview with Case 1 was not possible. No direct environmental measurements, radon testing, household water analysis, biomonitoring, indoor air assessment, or formal occupational hygiene evaluation were available. Germline testing was not performed in either patient. ATRX immunohistochemistry was not included in the initial diagnostic panel of Case 1; it was incorporated at recurrence. The +7/−10 chromosomal signature was not assessed in either case. The exposure questionnaire was administered to a single informant during acute clinical and psychological stress; information provided on behalf of the deceased partner carries inherent recall bias and informational incompleteness that cannot be corrected retrospectively (40). The two tumors, while both IDH-wildtype WHO grade 4, belong to molecularly distinct subtypes; this report is therefore descriptive and hypothesis-generating.

## 5 Conclusion

Two unrelated adults sharing household and workplace developed glioblastoma, IDH-wildtype, within 20 months. The two tumors satisfy WHO 2021 criteria for CNS WHO grade 4 yet belong to distinct molecular subtypes: a pTERT-negative giant cell GBM with secondary gliosarcomatous evolution, and a pTERT-mutant GBM with biopsy-limited characterization. The divergent histomolecular profiles argue against a simplistic shared molecular trajectory, but do not exclude shared exposure, shared susceptibility, or chance concurrence. Structured retrospective exposure assessment identified no specific shared exposure. The contribution of this report lies in the depth of its molecular documentation, including longitudinal profiling at recurrence, and in an explicit accounting of what was and was not measured. We encourage investigators who encounter future suspected glioma clusters to adopt a standardized, transparent exposure-assessment framework of the kind presented here, pairing current molecular classification with an explicit inventory of measured and unmeasured domains, so that individual reports can accumulate into a comparable, interpretable evidence base rather than remaining isolated anecdotes.

## Data Availability

The datasets presented in this study can be found in online repositories. The names of the repository/repositories and accession number(s) can be found below: zorodo.
