# Supplementary material for: Case Report: Glioblastoma in cohabiting partners: a molecularly characterized temporal cluster
Source: Front Oncol. 2026 Jul 28;16:1890635. doi: 10.3389/fonc.2026.1890635 (PMC13457150; doi:10.3389/fonc.2026.1890635)
Supplement: Supplementary file 1 [file Table1.docx]

**Supplementary Table 1. *Structured questionnaire for retrospective clinical and exposure-history collection.***

Administered to Case 2 during the diagnostic hospital admission preceding stereotactic biopsy. Respondent answered for himself and, to the extent known, for his deceased partner.

| **§** | **Domain** | **Items assessed** |
| --- | --- | --- |
| A | **Demographic and cohabitation data** | Name; date and place of birth; sex; marital status; education level; current and previous occupations; current and all previous addresses (preceding 30 years); duration of cohabitation and any periods of separation; house type, construction materials, year of construction, and maintenance history; presence of mold, humidity, or unusual odors; heating and air-conditioning sources; household chemical products; pet ownership. |
| B | **Family and genetic history** | Known family cancers and age at diagnosis; hereditary tumor syndromes (Li-Fraumeni, Turcot, neurofibromatosis); genetic testing results; other neurologic diseases in the family. |
| C | **Personal clinical history** | Date of glioma diagnosis and presenting symptoms; previous tumors or neurologic diseases; chronic diseases; previous diagnostic or therapeutic radiation exposure; regular medications; known allergies; vaccination history. |
| D | **Environmental history** | Residential history (preceding 30 years): duration, address, housing conditions; known hazardous materials (asbestos, lead); mold, humidity; heating type and fuel; household chemicals; active and passive smoke exposure; proximity to external pollution sources; EMF sources near the home; personal electronic-device use; known drinking-water analyses. |
| E | **Occupational history** | Complete work history (preceding 30 years): dates, job titles, tasks; documented exposure to toxic substances (asbestos, solvents, pesticides, ionizing radiation, heavy metals); use of personal protective equipment; acute or chronic exposure incidents; workplace EMF sources. |
| F | **Lifestyle and habits** | Usual diet; alcohol use; active and passive smoking; physical activity; recreational substance use; travel or residence in areas with reported environmental risk; animal contact. |
| G | **Clinical and molecular tumor data** | Histologic type and subtype; molecular genetic alterations; MGMT promoter methylation status; imaging studies; treatments (surgery, chemotherapy, radiotherapy, response); blood tests or biomarkers. |
| H | **Specific environmental exposures** | Home radon measurements and results; available indoor and outdoor air-quality data; documented exposure to environmental carcinogens; measured or estimated EMF; extraordinary environmental events (chemical incidents, fires, floods). |
| I | **Documentation and materials** | Availability of complete medical records; preserved biological samples (tissue, blood); genetic and molecular reports; toxicologic or environmental reports; known epidemiologic studies or reported tumor clusters in the area. |
| J | **Additional notes** | Any other potentially relevant detail or observation not captured by the above sections. |

**Supplementary Table 2*. Comparison among published reports of gliomas in spouses or cohabiting partners.***

Reports are presented as illustrative comparisons rather than as a systematic review. The Fehr and Auer (2022) case involved individuals who had been separated for 20 years and is categorically distinct from cohabiting pairs.

| **Report** | **Relationship** | **Interval** | **Tumor type(s)** | **Molecular data** | **Interpretation** |
| --- | --- | --- | --- | --- | --- |
| Tupchong et al. (1985) | Married cohabiting spouses | 18 months | Grade 2 gemistocytic astrocytomas (both) | Not available | Shared environmental factor postulated |
| Griffin et al. (1986) | Married cohabiting spouses | <2 years | Glioblastoma multiforme (both) | Not available | Etiology unresolved |
| Roviello et al. (2013) | Married cohabiting spouses | ~1 month | Glioblastoma multiforme (both) | Limited IHC only | No causal exposure demonstrated |
| Fehr & Auer (2022) | Divorced; not cohabiting for 20 years | Same day | High-grade IDH-wt diffuse glial tumors | Molecular data reported | Independent tumorigenesis favored |
| Present report | Unrelated adults; shared household and workplace | ~20 months | Giant cell GBM (Case 1) + IDH-wt HG glioma with WHO 2021 features (Case 2) | Full molecular characterization including recurrence profiling (Case 1) | Temporal cluster; divergent molecular subprofiles; etiology unresolved |
